# Detailed Evaluation of Possible Ganglion Cell Loss in the Retina of Zucker Diabetic Fatty (ZDF) Rats

**DOI:** 10.1038/s41598-019-46879-1

**Published:** 2019-07-18

**Authors:** Rozina I. Hajdú, Lenke K. Laurik, Klaudia Szabó, Bulcsú Dékány, Zsuzsanna Almási, Anna Énzsöly, Arnold Szabó, Tamás Radovits, Csaba Mátyás, Attila Oláh, Ágoston Szél, Gábor M. Somfai, Csaba Dávid, Ákos Lukáts

**Affiliations:** 1 Department of Anatomy, Histology and Embryology, Semmelweis University, Budapest, Hungary; 2 Department of Ophthalmology, Semmelweis University, Budapest, Hungary; 3 Heart and Vascular Center, Semmelweis University, Budapest, Hungary; 4 Retinology Unit, Pallas Kliniken, Olten, Switzerland

**Keywords:** Diabetes complications, Retina

## Abstract

A thinning of the inner retina is one of the earliest potential markers of neuroretinal damage in diabetic subjects. The histological background is uncertain; retinal ganglion cell (RGC) loss and changes in the structure or thickness of the inner plexiform layer (IPL) have been suspected. Studies conducted on animal models on RGC pathology gave contradictory results. Hereby we present RGC numbers, distribution patterns and IPL thickness from Zucker Diabetic Fatty (ZDF) rats. After labelling RGCs on retinal whole mounts, isodensity maps were constructed, RGC numbers and distribution patterns analysed using a custom-built algorithm, enabling point-by-point comparison. There was no change in staining characteristics of the antibodies and no significant difference in average RGC densities was found compared to controls. The distribution patterns were also comparable and no significant difference was found in IPL thickness and stratification or in the number of apoptotic cells in the ganglion cell layer (GCL). Our results provide a detailed evaluation of the inner retina and exclude major RGC loss in ZDF rats and suggest that other factors could serve as a potential explanation for inner retinal thinning in clinical studies. Our custom-built method could be adopted for the assessment of other animal or human retinas.

## Introduction

Diabetes mellitus is a major burden on societies not only in developed countries but also in the developing world with the costs of the treatment of diabetic complications constantly on the rise^1^. Indeed, the leading cause of legal blindness in the working-age population of developed countries is diabetes, through proliferative diabetic retinopathy and diabetic macular oedema^2,3^. The sequel of this latter two conditions are relatively well understood, being mainly capillary non-perfusion (leading to retinal ischemia) and vascular leakage (leading to retinal oedema and consequent photoreceptor loss)^4–6^.

There is somewhat less information on the course of early retinal changes in diabetes in humans. Clinical evidence suggests that there is a loss of retinal ganglion cells (RGCs) preceding the manifest retinal changes, before detectable retinopathy occurs^7–10^. The loss of contrast sensitivity^11,12^ and decreased response on electroretinography have already been described earlier^13–18^. There is a relatively strong body of clinical evidence with optical coherence tomography (OCT) suggesting a loss of inner retinal structures, namely the ganglion cell layer (GCL) and inner plexiform layer (IPL) complex with some involvement of the retinal nerve fiber layer as well^19–23^.

An extensive series of animal studies have been conducted to try to reveal retinal ganglion cell pathology in diabetes and address the above problem of early retinal changes. However, the results of these studies are contradictory as some studies show evident involvement of the RGCs^8–10,24^ while others do not^25–27^.

The importance of these findings lies in the understanding of the early sequelae in retinal pathology leading to the severe late complications. It has been postulated that the observed inner retinal loss corresponds to retinal neurodegeneration that is a prequel to later DR stages^10,28^, while on the other hand it could potentially serve as a surrogate marker for diabetic neuropathy (DNP)^29^. In the case of DNP it is very difficult to find reliable biomarkers and therefore the above clinical observations could serve as clinical surrogates in the diagnosis and therapy of DNP. One further implication of this theory of diabetic retinal neurodegeneration could serve as a link between the suspected increased prevalence of glaucoma, that is, progressive optic neuropathy, and diabetes^30,31^.

Previously, our group has conducted a study on Zucker Diabetic Fatty (ZDF) rats, a well-known model of type 2 diabetes mellitus^8–10,24,27^. Utilizing retinal sections only, we were able to give a general overview of almost all cell types of the retina, and managed to identify a set of pathologic alterations (glial response, photoreceptor degeneration, alterations in some amacrine cell numbers) in diabetic ZDF rats. However, based on the Brn-3a stained sections we could not confirm any change in the ganglion cell numbers.

Due to the huge potential importance of RGCs in understanding early diabetic retinal pathology, the contradictory results on other experimental animals^25–27,32^ and the uncertainty of analysing RGC loss based on a relatively small number of sections only, we decided to conduct a more detailed assessment in the same model, using whole mounted complete retinas, three different pan-ganglionic markers, and isodensity maps analysed with the help of a custom-built algorithm that was developed to compare isodensity maps point-by-point. Additionally, IPL thickness and stratification were also examined.

## Results

### Body weights and blood glucose levels

The time-course of body weight changes of the ZDF rat strain vs. lean controls were similar to those published by the supplier (Charles River Laboratories, Sulzfeld, Germany). There was no significant difference in body weights detectable at the time of anaesthesia at the 32^nd^ week (421.3 ± 21.6 g in lean and 400.3 ± 50.2 g in diabetic animals).

**Figure 1:**
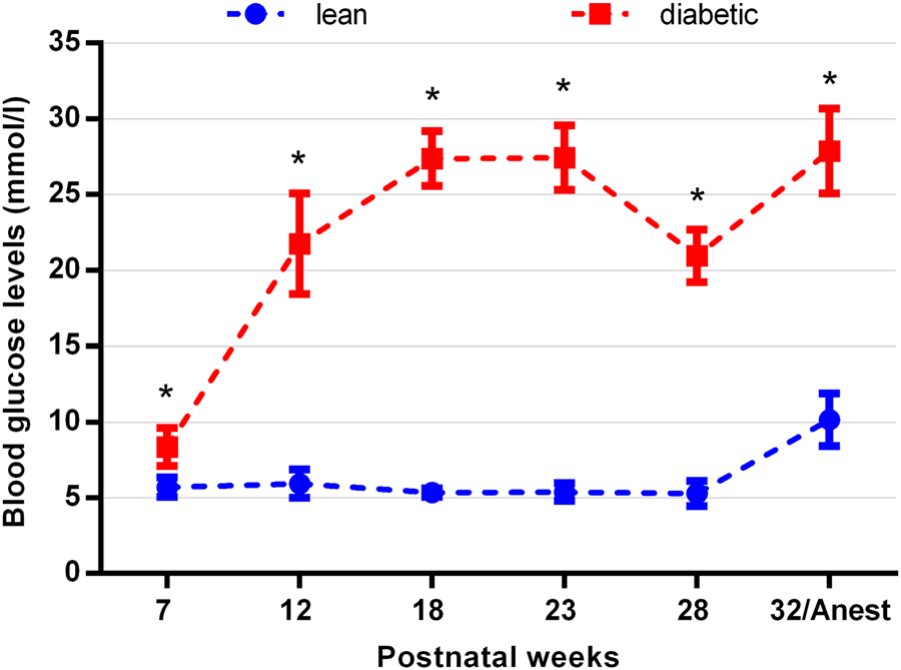
Blood glucose levels in diabetic and lean ZDF rats. Blood glucose levels were significantly higher from postnatal week 7 till the end of the observation period. *p < 0.05. The figure and the detailed statistical analysis has already been published in a previous work^27^.

Blood glucose levels were already significantly higher at the 7^th^ week and remained elevated throughout the complete observation period in diabetic animals. In lean controls blood glucose levels remained normal, except for values measured during anaesthesia(Fig. 1). Details about the statistics with the appropriate P values for each time point have already been given in another report^33^.

### Triple retinal ganglion cell labelling on cryosections

**Figure 2:**
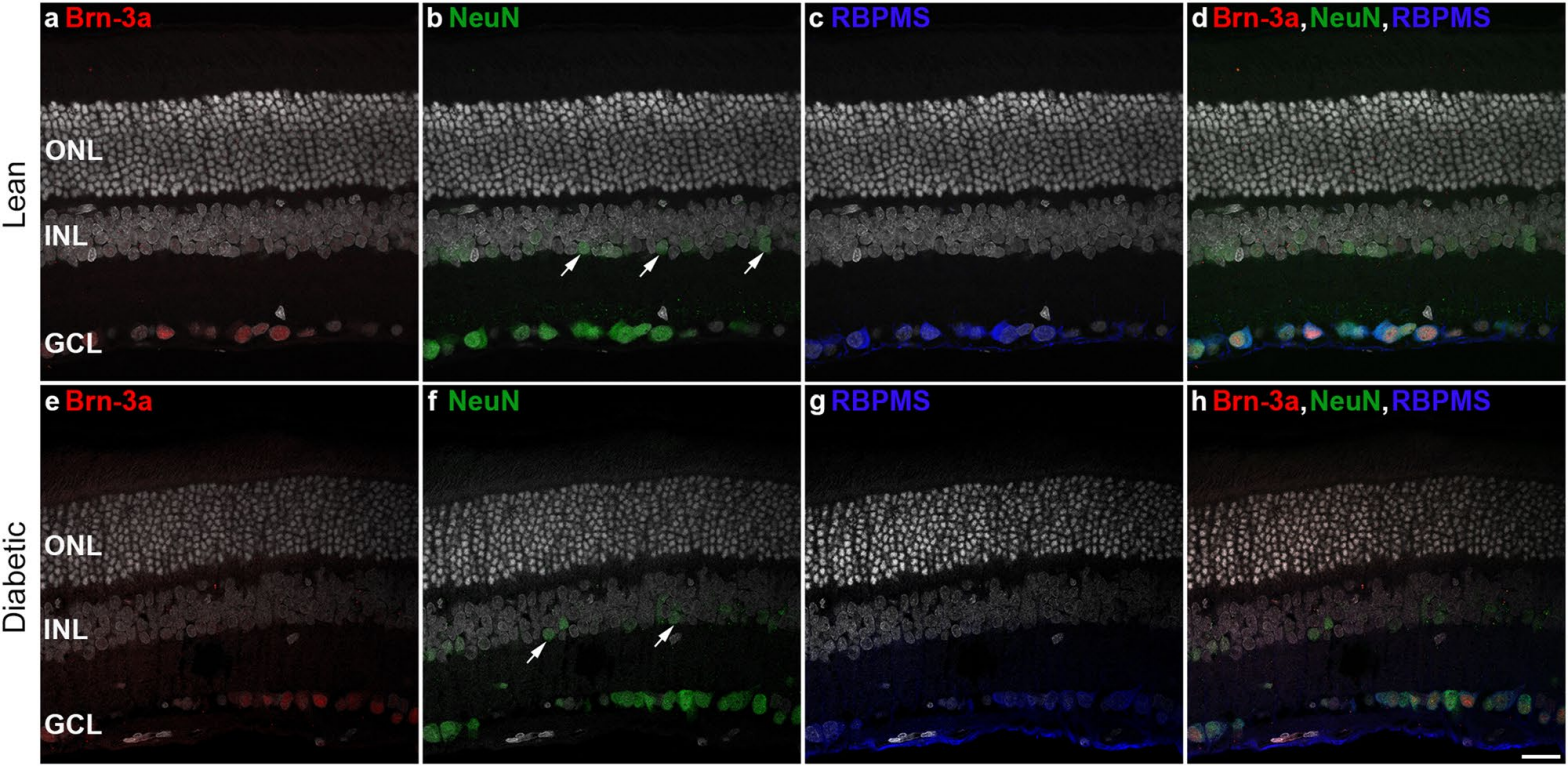
Triple labelling of retinal ganglion cells in retinal cryosections. Representative images of retinal sections of control lean (**a**–**d**) and diabetic (**e**–**h**) specimens labelled by three different markers Brn-3a (first column - in red), NeuN (second column - in green) and RBPMS (third column – in blue). Merged images are shown in the right column. The vast majority of the RGCs are labelled by all three markers. NeuN also labels a population of cells – most probably amacrine cells - in the INL (arrows on **b**,**f**). DAPI is used as a nuclear staining on the sections (in white). ONL: outer nuclear layer, INL: inner nuclear layer, GCL: ganglion cell layer. *Bar*: 20 µm.

Three antibodies were used in the study to label the majority of RGCs (Fig. 2). In agreement with literature data, Brn-3a staining showed a purely nuclear localization^34^, NeuN besides labelling the nuclei also stained the cytoplasm of the cell^35^ whereas RBPMS^36^ positivity was a prominently located in the cytoplasm. The vast majority of the labelled cells in the GCL were stained by all three antibodies; only occasionally did we find cells positive for two stains or one single stain only. Besides the staining pattern in the GCL, NeuN also detected a relatively large number of smaller cells in the inner nuclear layer (INL - most probably amacrine cells – arrows in Fig. 2b,f) that did not co-label with the other two antibodies used. Larger cells in the INL were also visible regularly, albeit in an extremely small number, those were usually positive for all three antibodies. Literature data suggest that they are displaced retinal ganglion cells (Supplementary Material Fig. S1)^35,37^.

Comparing the control and the diabetic specimens, no major difference was evident in the staining intensity or in the number and localization of the labelled elements with any of the antibodies used. We did detect, however, a prominent variation between retinal regions, especially in the central retina, adjacent to the optic nerve head. In some parts, no labelled cells were present while merely 20–50 µm away, a large group of cells were detectable. This phenomenon was clearly visible in both control and diabetic specimens.

### Triple ganglion cell labelling on whole mounted retinas

**Figure 3:**
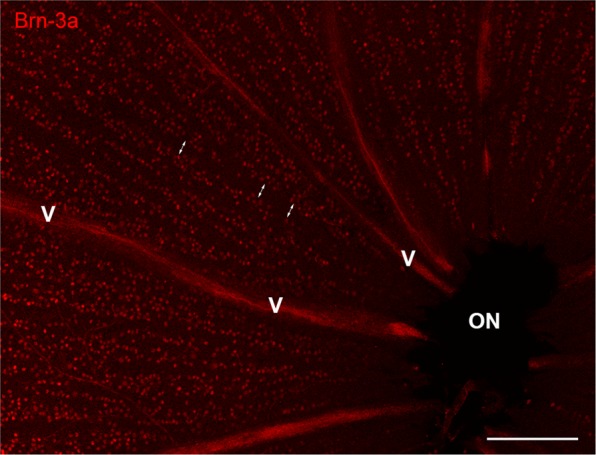
Uneven retinal ganglion cell distribution near the optic nerve head. Representative image of Brn-3a labelled RGCs in the region of the optic nerve head (ON). The vessels (V) and trunks of ganglion cell fibers (double arrows) are free of ganglion cell nuclei. ON: optic nerve head, V: vessels. *Bar*: 200 µm.

On whole mounted retinas, similarly to sections, the majority of RGCs was labelled by all three antibodies in both groups. Due to the cytoplasmic stain of the NeuN and RBPMS antibodies, precise quantification was difficult to perform with these two labels in the central retinal regions. Near the optic nerve head, the axons of ganglion cells collect into broad bundles pushing RGCs apart. Furthermore, the major vessels radiating from the optic nerve head are also free of RGC bodies, which are forming tightly packed columns in between the axon bundles and vessels (Fig. 3). This uneven distribution can potentially account for the irregularity observed in cryosections and must be taken into account during quantitative analysis. Choosing a relatively small counting frame on whole mounts would result in either positive or negative errors in calculating RGC densities in the central retina.

Overlapping cell bodies make it difficult if not impossible to reliably count with NeuN or RBPMS in the central retina. Precise quantification, however, remains possible with the nuclear stain of Brn-3a.

**Figure 4:**
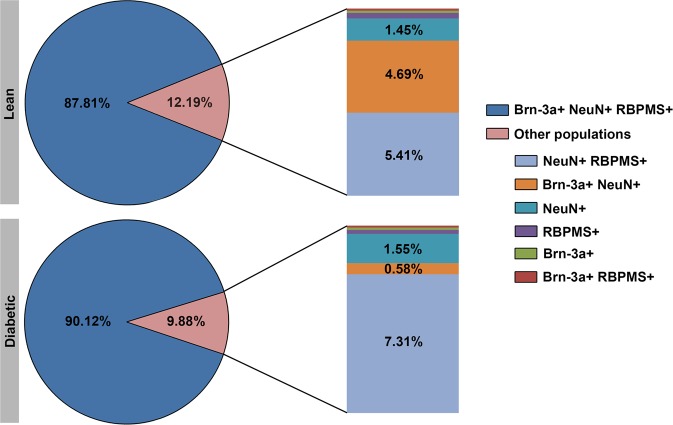
Quantitative analysis of the RGC populations performed on whole mounted retinas. The palette of three different kinds of pan-ganglionic stainings (Brn-3a, RBPMS and NeuN antibodies) were applied. The majority of RGCs was stained by all three antibodies. The detailed quantification of the remaining double or single positive populations are given on the right side of the diagram. The numbers indicate the percentages within the total RGC population. Some populations were present in negligible amount only (RBPMS+: 0.36% vs. 0.21%; Brn-3a+: 0.14% vs. 0.13%; Brn-3a+ RBPMS+: 0.14% vs. 0.11% in lean and diabetic specimens respectively). No major difference was evident between lean and diabetic specimens.

To estimate the number of cells labelled by the other two antibodies (NeuN and RBPMS), we analysed the staining characteristic of the individual RGCs in the peripheral and mid-peripheral counting frames (with the central region excluded) against the panel of the three antibodies used and the results were expressed as percentage of all detected RGCs. As clearly indicated by these results summarized in Fig. 4, the vast majority of the cells were labelled by all three antibodies in both groups (87.81% in control vs. 90.12% in diabetic specimens) and there was no robust change detectable in the values between diabetic and lean rat retinas. These results in agreement with those obtained from triple labelled sections clearly demonstrate, that the results of Brn-3a counts reliably represents the majority of the RGC population and none of the antibodies showed a decrease in staining intensity.

Taking it all into account, in this report we present the results of the precise quantification of Brn-3a labelled RGCs only, with the counting performed in frames of 200 µm × 200 µm in size.

**Figure 5:**
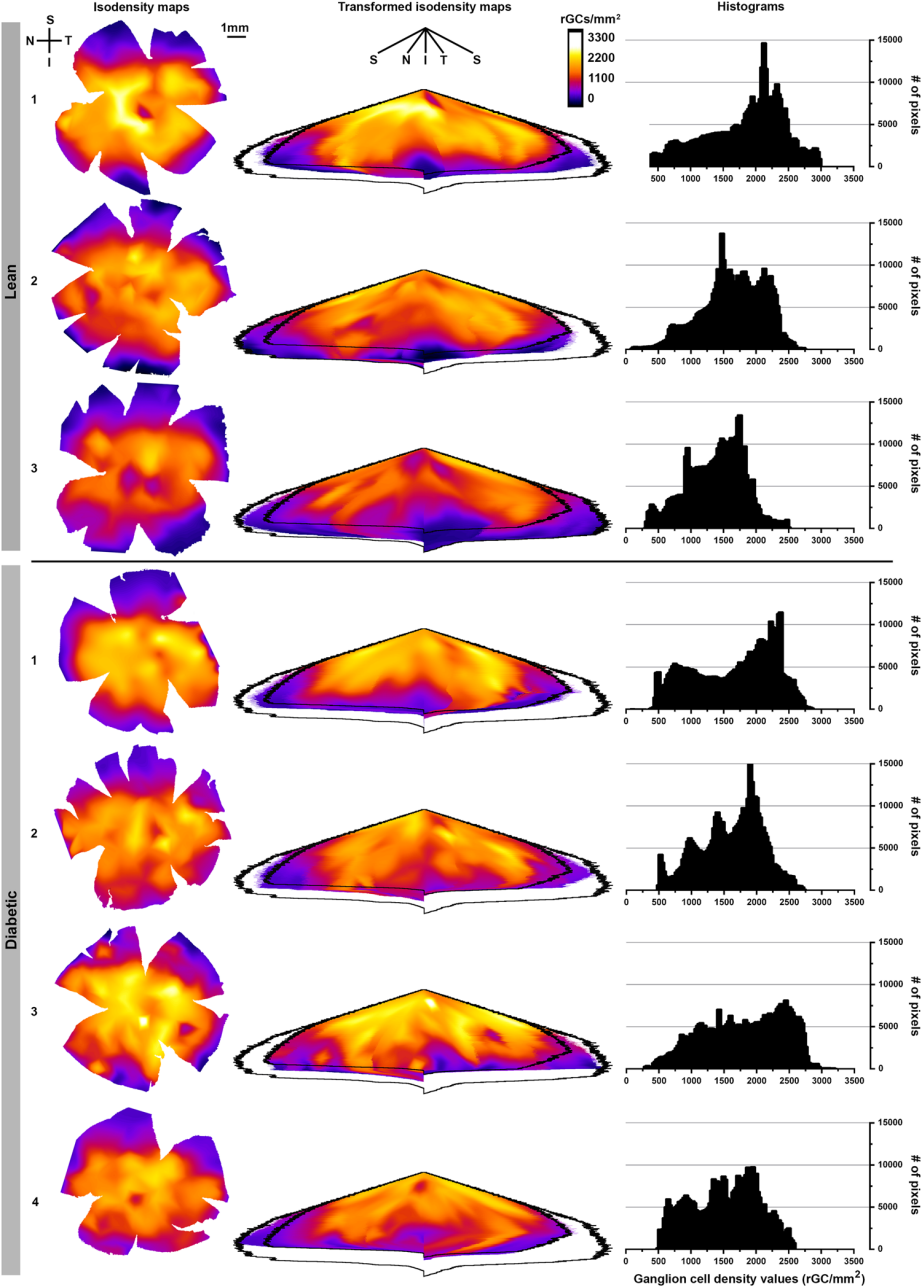
Comparison of retinal ganglion cell distributions in lean controls and diabetic specimens. Colour-coded isodensity maps (left column) from 3 control and 4 diabetic retinas. The colours indicate different RGC density values. Similar distribution patterns were detectable in both groups. To maximize overlapping regions the retinal maps were transformed as described in detail in the materials and methods section and shown on Fig. 6. The resulting transformed maps are presented in the middle column, where the overlapping regions are marked by the inner-, and the theoretical ideal retina - with absolutely no region lost during preparation -by the outer black contour. The difference between the area of the actual map and the outer black contour is the “loss during preparation” that seems to be greater in the diabetic group. No major difference in cell densities is evident between the two groups in the overlapping region. Right column: histograms of retinal ganglion cell density values. Data are given in ganglion cell numbers/mm^2^. The results of quantitative statistical analysis for the complete maps and also for the overlapping region are given in Table 1. S: superior, I: inferior, N: nasal, T: temporal. *Bar*: 1 mm.

The results of RGC densities estimated from Brn-3a labelled retinas are summarized in Fig. 5 and Table 1. The constructed isodensity maps shown in Fig. 5, left column, revealed similar RGC distribution patterns in both lean controls and diabetic specimens. A clear, but slightly asymmetric centro-peripheral gradient could be observed in both groups. Central and mid-peripheral density values were in the range of 1500–3000 RGCs 1/mm^2^, which dropped to values around 500 RGCs 1/mm^2^ in the peripheral retina, with lower values in the superior retinal half. There was some variation between specimens even from the same group but no evident difference was visible between the control and the diabetic groups. The isodensity maps were used to calculate average density values and the retinal areas were also estimated. The results shown in Table 1 demonstrate that the average density values calculated for the total retina were slightly higher in the diabetic group (1562.00 RGCs 1/mm^2^ in diabetic vs. 1415.15 RGCs 1/mm^2^ in control retinas) while the average total retinal area was slightly smaller in diabetes (51.90 mm^2^ in diabetes vs. 59.78 mm^2^ in controls), but the differences were not significant (p = 0.14 and p = 0.97 for ganglion cell densities and for retinal areas, respectively). In order to compare these results with published literature data from control rats^37,38^, we also calculated the estimated total RGC numbers for all retinas.

**Table 1:** Results of the quantitative analysis of the isodensity maps.

		Estimated total number of RGCs per retina	Total retina average RGC density (RGCs/mm^2^)	Overlapping area average RGC density (RGCs/mm^2^)	Area of the total retina (mm^2^)	Loss during preparation (mm^2^)
Lean controls	1	90670.44	1634.88	1808.08	55.46	11.04
	2	75753.57	1175.75	1381.65	64.43	2.07
	3	85286.30	1434.83	1639.63	59.44	7.06
	average	84597.67	1415.15	1609.78	59.78	6.72
Diabetic	1	81034.79	1521.78	1614.15	53.25	13.25
	2	90279.99	1683.70	1804.30	53.62	12.89
	3	72689.57	1476.83	1521.08	49.22	7.28
	4	80617.89	1565.70	1667.25	51.49	15.01
	average	81067.80	1562.00	1651.69	51.90	14.61

A detailed description of how the total and overlapping retinal areas, density values and the suspected loss during preparation was estimated can be found in the Materials and methods section.

**Figure 6:**
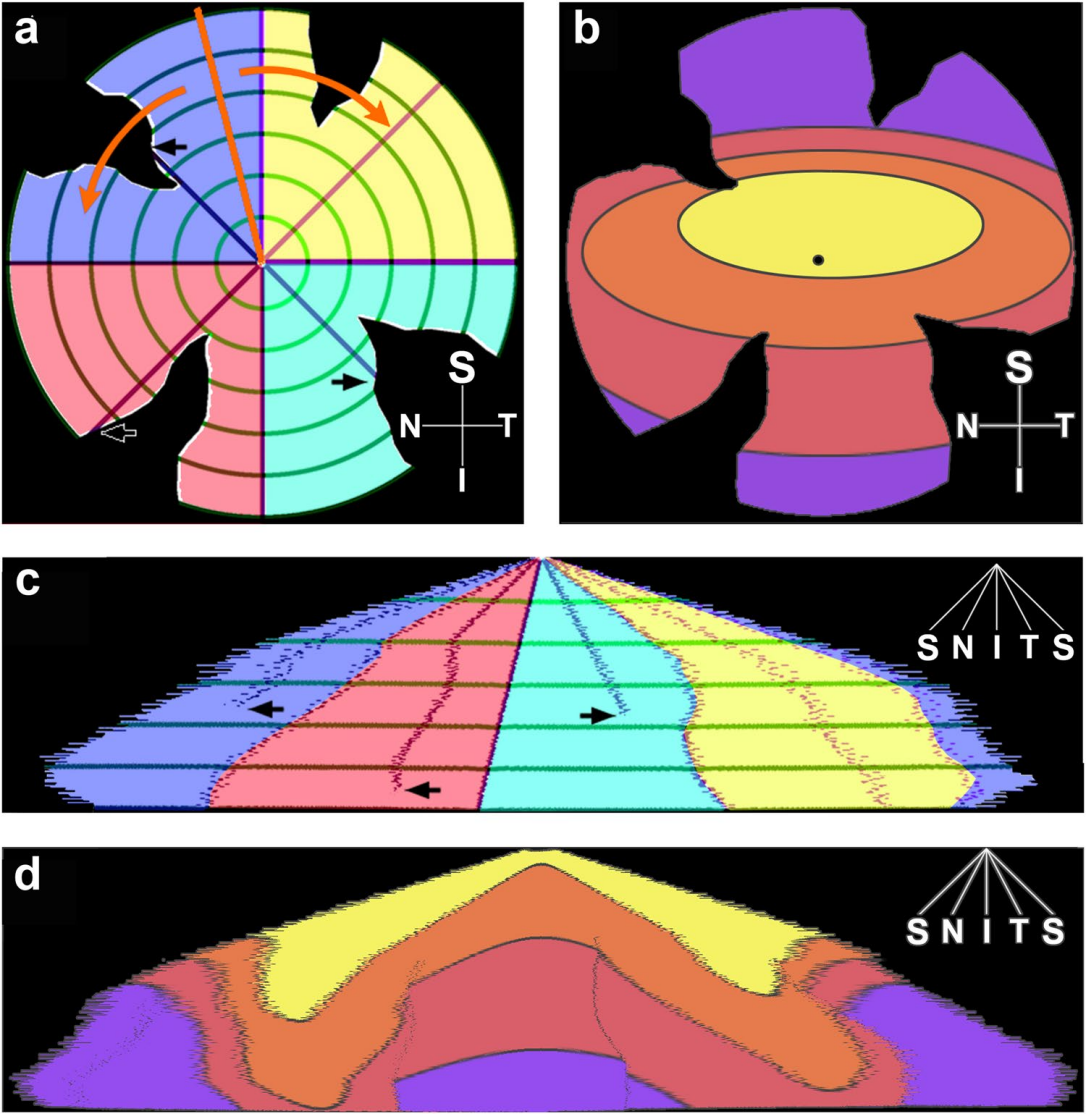
Transformation of the whole mount retinas. Schematic drawing of the original whole mount of a retina with the four quadrants labelled by different colours (**a**) showing radial (purple and blue lines) and concentric (green circles) guide lines projected on it. The dorsal direction is marked with an orange guide line. The distance from the optic disc and the angle from the orientation guide line was calculated for each pixels of the retina. Then each pixel was repositioned according to their angle (x-axis) and distance (y-axis) which is equivalent of opening the retina along the orange guide line, and bend it down along (orange arrows on **a**) similar to close a hand fan halfway. The resulting image (**c**) shows the originally concentric green guide line as horizontal lines. The purple radial guide lines that hit an incision do not reach the baseline (**a**,**c**, black arrows), i.e. the perimeter of the retina. Note that inside of the first two circular guidelines (green) the purple lines are linear, but then they were bent due to the excluded pixels of incisions. Colour-codes represent the original position of the pixels on the retinal whole mounts. For comparison with conventional retinal heat maps, an ideal flat-mounted retina is also displayed (**b**), where colour codes represent different density values. (**d**) Depicts the same ideal retina after the transformation. S: superior, I: inferior, N: nasal, T: temporal.

Due to the cuts made in order to flatten the retinas, that are located in slightly different positions, the overlapping area of the retinas is relatively small, making point to point quantitative comparison difficult. In order to overcome this problem, the retinas were transformed as detailed in the Materials chapter by a custom-built algorithm to eliminate cuts. A schematic drawing about how this transformation was performed is given in Fig. 6, for better understanding. The resulting transformed maps are shown in Fig. 5, middle column. These transformed maps allowed us to estimate the size of the ideal retina (with absolutely no loss of peripheral regions – see the Materials and methods section for details), giving a total of 66.50 mm^2^. The transformation also allows us to calculate and maximize the overlapping region for all retinas analysed, which was 46.06 mm^2^. Two contours, the ideal and overlapping areas were created, projected onto the maps and marked by black outlines. Average density values were recalculated for the overlapping regions only (Table 1) but again revealed no significant difference between control and diabetic groups (1651.69 RGCs 1/mm^2^ in diabetic vs. 1609.78 RGCs 1/mm^2^ in control specimens, p = 0.4). The difference in area between the ideal retinas and the individual maps were also quantified for each retina, and this “loss during preparation” was significantly higher in diabetic specimens (Table 1, 14.61 mm^2^ in diabetes vs. 6.72 mm^2^ in controls, p < 0.0001). Given the fact that RGC density values are practically identical in the overlapping region, the higher peripheral loss of retinal sample can potentially account for the slightly higher density values detected in the complete diabetic retinas (total retina, average RGC density).

**Figure 7:**
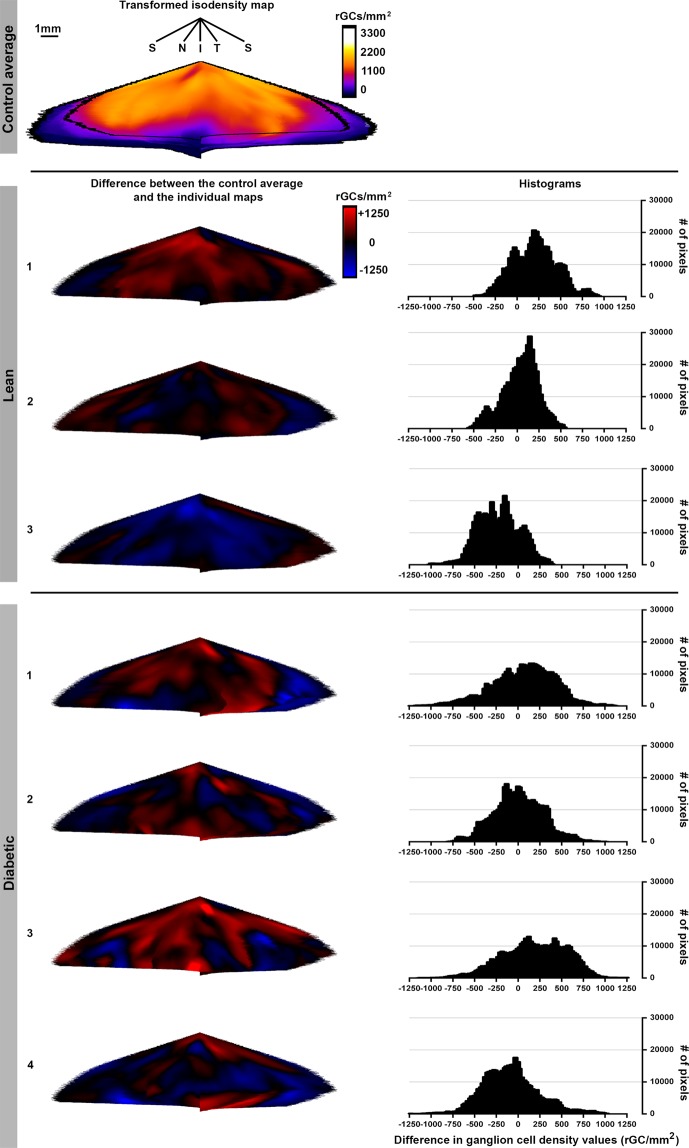
Regional differences in retinal ganglion cell distribution patterns in lean and diabetic rat retinas. An average control isodensity map was created from the transformed retinal maps as described in the materials chapter (upper row). This map could be compared with the individual transformed isodensity maps. The resulting difference maps (only created for the overlapping regions) are shown below. Red colour indicates areas with higher, blue colour with lower density values. The results are also show in a form of histograms (right column). Note that there was no evident trend in the direction of change and the areas involved. Density values are given in RGC/mm^2^. S: superior, I: inferior, N: nasal, T: temporal. *Bar*: 1 mm.

Although average density values showed no difference in diabetic specimens compared to controls, this fact does not automatically exclude potential regional differences between retinas of different groups. In order to quantify regional differences, an average transformed RGC map was created from the control retinas (“average control retina” shown in Fig. 7) and the individual retinas were subtracted from it. Details about this transformation can be found in the Materials and methods section. The resulting difference maps for each retina and the histograms are demonstrated in Fig. 7. Although – in agreement with literature data^39,40^ – there is a relatively high individual variation even in the retinas from the same group, no consequent trend could be observed between the two groups for any region examined.

### Apoptosis amongst ganglion cells

The average number of TUNEL positive cells in the GCL per each vertical section were 1.38 ± 1.54 in control vs. 1.26 ± 1.24 in diabetic specimens. There was no significant difference detectable (p = 0.73). Representative images of TUNEL labelled control and diabetic retinas have been published in the Supplementary material of our previous article^41^ and are also given in the Supplementary materials (Supplementary Fig. S2).

### IPL thickness

**Figure 8:**
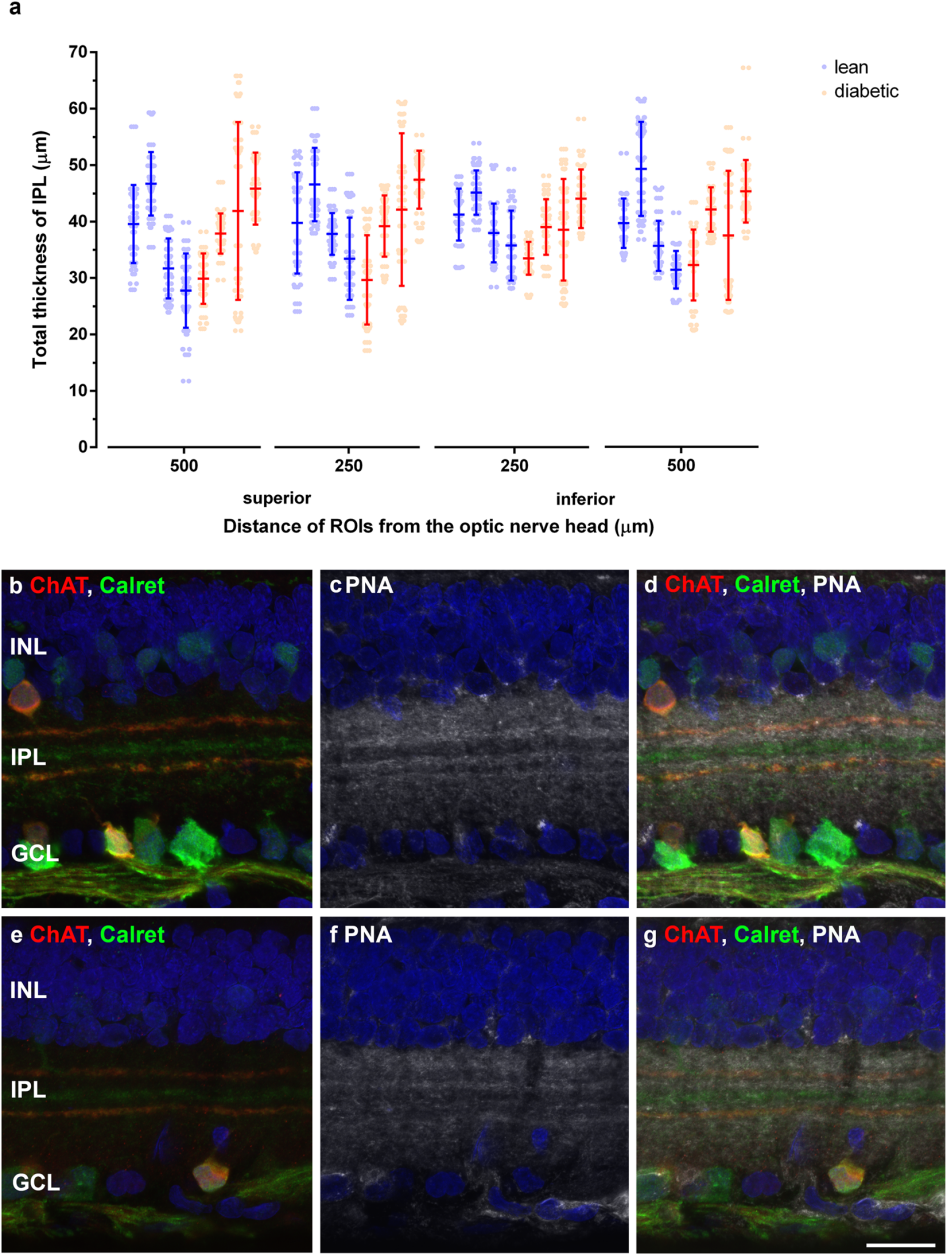
Thickness and stratification pattern of the IPL in control and diabetic retinas. Total IPL thickness was measured at four different central locations (250 and 500 µm superiorly and inferiorly from the optic nerve head) in four specimens from each group. Results from each specimen are plotted individually (**a**). Despite the great standard deviations within the same specimens and within the same group, no evident change was seen in between control and diabetic groups. The average values in different positions given in µm (mean ± SD) and the appropriate P values were: superior 500 µm lean: 36.42 ± 8.42 vs. diabetic: 38.87 ± 6.82p = 0.67; superior 250 µm: lean: 39.76 ± 5.74 vs. diabetic: 39.59 ± 7.45 p = 0.96; inferior 250 µm lean: 40.02 ± 4.09 vs. diabetic: 38.77 ± 4.32 p = 0.44; inferior 500 µm lean: 39.04 ± 7.64 vs. diabetic: 39.33 ± 5.68 p = 0.96. Stratification of the IPL was assessed on cryosections using calretinin (b, e - in green), ChAT (**b**,**e** - in red) and PNA stainings (**c**,**f** – in grey). Merged images: (**d**,**g**). Nuclei were counterstained by DAPI (**b**–**g** - in blue). No evident change was detectable. INL: inner nuclear layer, IPL: inner plexiform layer, GCL: ganglion cell layer. *Bar*: 20 µm.

The thickness of the IPL was estimated on four different centrally located regions. Results are shown in Fig. 8. Due to the uneven border of the IPL towards the INL and GCL we detected a relatively high standard deviation in IPL thickness in all counting positions, in both groups and relatively large differences were seen amongst specimens of the same group, but no significant difference was detected between lean and diabetic specimens at any positions assessed.

### IPL stratification

Calretinin^42,43^, ChAT^24^ and PNA lectin labeling^44^ used were able to visualize certain sublayers of the IPL (Fig. 8). Comparing the number and positions of the layers detectable, no evident difference emerged, indicating that there was no major change in the stratified organization of the IPL.

## Discussion

Animal and human studies congruently suggest that both neural and vascular elements of the retina are damaged by the diabetic environment. Neural and vascular retinal tissue are damaged as a complex unit: alterations in early diabetes (preceding clinically manifest vasculopathy) include changes of certain characteristics of neural cell types including morphology, number, neurotransmission, synapses, as well as subclinical vascular changes. Amongst neural cell types the involvement of photoreceptor cells^8,45–47^, horizontal, amacrine^8,9,24,48^ and retinal ganglion cell types^19–23^ has been documented. It is still obscure though how the neurodegeneration and vasculopathy are related to each other, whether or not the vascular of neural changes trigger each other. There is a general agreement, however, that damaged cells – both neurons and vascular cells - will eventually die, leading to a progressive cell loss in diabetes.

Apoptotic loss of neural as well as vascular elements has been reported in many different animal models^28,49^. Furthermore, postmortem human samples and *in vivo* OCT data show evidence that progressive cell death may eventually lead to the thinning of the retinal layers. Specifically, the inner retinal layers and ganglion cells are thought to be the most vulnerable^50^. According to some human OCT data, loss of ganglion cells may be the first morphological sign of diabetes, preceding clinically detectable vasculopathy^51,52^ or even present in metabolic syndrome^53–55^. With the recent introduction of OCT Angiography (OCTA) it is possible assess the *in vivo* capillary structure of the macula. It seems that the microvascular and retinal changes are coexisting and appear to be well detectable^10^.

However, possibly due to the different methods and different study settings, OCT studies are inconsistent and not all studies confirm neurodegenerative thinning of the inner layers^28^. It is also very important to note that most studies showing *in vivo* “thinning” are mostly case-control studies, comparing two, matched groups in terms of morphology. That is, no real “thinning” of the inner retina but thinner structures between two populations are being described at a given time point. In a 4-year longitudinal study of 41 T1DM subjects, 18 patients demonstrated the progression of DR and developed a progressive loss in NFL and GCL + IPL^48^. On the contrary, a recent study with a 3-year follow-up in 91 T2DM subjects showed no changes in the inner retina; on the contrary, the outer retina seemed to be significantly involved in changes over time^53^.

Similarly to human data there is a lot of discrepancy also in different animal models concerning the timing of cell death and the sensitivity of specific cells to hyperglycaemia. Even within the same model – like in ZDF rats used in the present study – controversial data has been published. Some reported an increase in the number of apoptotic cells already in 19 week-old ZDF rats^48^ while in another report on 23 week-old animals by Johnson and co-workers^25^ and also in a previous work of our group on 32 week-old rats^56^ no cell loss was observed. The reason behind this discrepancy is currently unknown but due to its huge potential impact on diagnosis and therapy it calls for further studies. In order to undoubtedly prove or exclude whether RGC loss is present in ZDF rats, we conducted a more detailed evaluation of inner retinal pathology in specimens of the same experiment^27^. The current report also presents strong evidence against early RGC loss in ZDF rats Several points need to be considered as potential explanations for the differences between these reports. They may be grouped into three major categories and are discussed below.

1. Variations in the detection method and quantification used. Various techniques have been applied for the detection of RGCs and other cell types and for detecting apoptotic cells. The differences in antibody and apoptotic kit selection or in the quantification and statistical approach may be responsible for the deviations. Indeed, in our view the most significant achievement of the study presented here is that it successfully excludes most of the possible flaws of techniques applied previously.
2. Differences in models or animal care. Animal models only mimic some characteristics of human diabetic retinal pathology. There are inherent differences between the models – and also amongst human patients - in the kinetics of blood glucose level rise, lipid or blood pressure parameters and other concomitant laboratory parameters and also in animal care (eg. use of insulin) or diabetes control in patients that can all influence the results.
3. Changes in IPL thickness or stratification pattern may be responsible at least for the thinning of the GCL-IPL complex in clinical OCT examinations.

Several techniques can be used to label RGCs and detect apoptotic elements^32–34,57^. Unfortunately, practically all methods have limitations. Counting all cell nuclei in GCL is not specific enough, as a significant percentage of the cells located there are displaced amacrine or glia cells. Retrograde labelling can only detect ganglion cells projecting to the target area injected (eg.: lateral geniculate body) leaving a (sometimes rather large) part of the RGC population unlabeled^58^. Immunocytochemistry is probably the most sensitive and adequately selective method for identifying RGCs. Due to their diverse subtypes, all antibodies available to date label only a portion, some of them even the vast majority of the RGC population but never all RGCs^56^. There are also evident differences in sensitivity and labelling characteristics between the antibodies available. Brn-3a was reported to be very sensible to noxious insults. Labelling for example disappears from most RGCs within days after optic nerve dissection^32–34,57,59^. This however, does not inevitably indicate that the cells die that early. In another report on diabetic *Meriones shawi*, NeuN was proven to be the most sensible marker of RGC loss, while Brn-3a and RBPMS were only able to detect the loss over a longer timescale^26^. In order to minimize the effects of the antibody choice and sensitivity, in this report we also used three different antibodies, each alone known to label the majority of RGCs in control rats^60–66^- with the hope that in case of ganglion cell injury a change in the staining of at least one of the antibodies should be evident. This triple labelling allowed us to identify RGCs both in the central and in the peripheral retinal piece and to analyse quantitatively RGCs against the panel of all three antibodies. Our results demonstrate that the vast majority of RGCs was co-labelled by all markers used both in the control and in the diabetic group, with no major change attributable to the disease. Also, no major change in the staining intensity of any of the markers was detectable in diabetic specimens. Therefore, for detailed studies Brn-3a was chosen, which as a nuclear label was proven to be the easiest to quantify. It is worth mentioning, however, that the polyclonal Brn-3a used here produced a more robust staining compared to its monoclonal counterpart used by us in previous studies^27^, that may explain this unexpected reliability and further underlies the importance of the proper antibody choice.

Quantification of RGCs may be performed on sections as well as on whole retinas or retinal pieces with plenty of examples for each technique in literature available^56^. The sampling method may be especially important in the central retinal regions, where RGC are not evenly distributed but are grouped into rows, with the regions of axon bundles and bigger vessels completely free of perikarya. Including only selected (even randomly selected) relatively small regions of the sections for quantification may thus lead to over- or under sampling of RGC density values if the number or regions analysed is not extremely large. On the other hand, if this uneven distribution pattern is taken into account, RGC density changes can be reliably predicted even from sections. Our previous study^27^ focusing on the general pathological features of diabetic ZDF rats a less detailed estimation of RGC loss was attempted on sections, using the Brn-3a antibody only, and produced practically the same results. Also, in another report published by Hammoum *et al*.^56^ utilizing both sections and whole mounted retinas RGC loss was similarly predictable using both methods.

Whole mounted retinas have one obvious advantage over sections: theoretically they are capable of detecting small regional differences restricted to certain retinal areas only. There are practically two distinct ways to quantify whole mounted retinas. Conventionally, RGCs can be identified and counted in randomly distributed sample fields of equal sizes while the values of uncounted regions can be estimated by interpolation^56,64^ and presented as isodensity maps. Alternatively, automatic cell counting procedures could also be used. A broad range of such techniques have been published to date^37,38,59^ which minimize human effort in whole mount retinal analysis and in theory are capable of reliably identifying practically all labelled ganglion cells. An obvious advantage over manual counting is that all (or almost all) ganglion cells are counted and the resulting maps could be analysed with much detail – assuming that ganglion cell identification was correct.

Automatic counting does have some important limitations as well. First, it may underestimate cell numbers in the central retinal regions, where ganglion cell density is the highest, and where the cells tend to group with overlapping profiles. Furthermore, possible errors may arise at the retinal edges and also around folds, tires and cuts – artefacts resulting from the mounting technique. This latter may be especially important in the case of diabetic retinas that are more subjected to injury both during preparation, immunocytochemistry and flatmounting. Taking this and the potential changes in staining intensity and pattern that may be detectable in diabetes into account, in order to minimize possible flaws of quantification, in this study the manual counting technique was chosen. Given the fact that the resulting density values and the calculated total ganglion cell numbers are in good agreement with those of other reports using automatic quantification^37,38,59^, this technique has also proved to be reliable.

Precise – ideally point to point - comparison of the resulting isodensity maps is also crucial in diabetes. In a previous report by our group^26^ a striking regional difference was observed in the rate of neurodegeneration in STZ-induced diabetic rats. Seemingly randomly distributed, smaller or larger degenerative patches altered with regions of normal or near normal structure. Furthermore, mfERG examinations of human diabetic patients indicate that the retina is not homogenously damaged by the disease^15^. The prerequisite for such comparison, however, is the near complete matching of identical regions from control and diabetic retinas. Although it seems to be an easy task, retinas always suffer from loss of peripheral regions during preparation, which is especially true for diabetic retinas. The reason for this is not exactly known, but most probably it is due to the increased protein glycation at the vitreoretinal border. This is in parallel with clinical observations as in diabetic patients the intraoperative active induction of posterior vitreous detachment is always pronouncedly more difficult compared to other pathologies^26^. Problems arise also from the uneven positioning of the cuts required to flatten the retina and from the fact that a near spherical 3D structure is transformed and presented in 2-dimensional maps. Several methods in literature have been published to overcome these problems^18^. Some – actually most – require sophisticated mathematical transformations and significant computational capacity. The method presented here is also capable to cope with most of the difficulties with comparing the retinas. It allows us to virtually remove cuts from the flat-mounts and transforms the retinas into an easy-to-analyse 2D structure, while preserving the orientation of the pixels of the original retina in relation to each other, without any loss of data during the transformation. Also, the process requires moderate computing capacity and further data processing can be performed by the freely accessible FIJI program pack. The maximum retinal area, the overlapping regions and the regional loss during preparation can be calculated easily. Minimum, maximum and average RGC densities can be estimated, compared and presented in pictorial form for any area of interest over a few clicks only. Using all the capacity of the algorithm, we examined the regional differences within the same group and between the diabetic and control groups. The results presented, although reveal a high individual variation in regional density values even within the same group, clearly exclude a tendentious RGC loss restricted to special regions only.

In line with the results of RGC quantification, in this study we found no increase in the number of TUNEL positive elements whether calculated for the complete retinal thickness (ONL-GCL) or just for the GCL alone. This contradicts a series of reports in literature stating that apoptosis is a relatively early process in the diabetic retina. In another study conducted in ZDF rats^48^ elevated apoptotic rate was detectable as early as 19 weeks of age. Currently we can only speculate about this contradiction. The reason behind this may lie in the technique itself. Although TUNEL is generally acknowledged as a selective apoptosis marker^67^, there has been a debate about its specificity from its introduction. It has been proven to label some of the necrotic cells^68^ as well as cells undergoing DNA repair^69^. Although significant efforts has been made to improve the technique, even today, manufacturers recommend to check nuclear morphology, or co-label with some other apoptotic marker (eg.: activated caspase-3)^70^ for the best results. Also, applying TUNEL staining on sections as was done here may not be the most sensitive method for detecting dying cells, if they are grouped into smaller regions. Whole mounted retinas or at least retinal pieces would be better to pick those damaged areas. Concerning RGCs however, when comparing individual maps with the average control maps allowed us to rule out relevant RGC loss in any region of the retina.

Differences in animal models and animal care may also be responsible for the different results of the studies published. A large variety of animal model systems of diabetes have been developed so far. They mimic some, but never all the features of human disease^71–74^. The models differ in the animal species and the induction method used (eg.: genetically or chemically induced DM), in the underlying metabolic conditions (rate of hyperglycaemia reached, hyperlipidaemia, insulin homeostasis), the presence or absence of other concomitant pathology (eg.: hypertension, obesity), the duration of maximum observation period and the rate of vasculopathy. Some of these factors, including hypertension and lipid homeostasis has been proposed to be a risk factor of DR^75–77^, therefore it would not be surprising if the models differed in the severity of retinal alterations. Interestingly in both T1D^26,78^ and T2D rat models^27^ studied by us with comparable methodology a similar rate of neurodegeneration was detectable and there was no difference in the type of cells involved. Also, there was no increase in apoptotic rate neither if estimated in all retinal layers or in the GCL specifically. Furthermore, as far as the RGCs are concerned, no decrease in their numbers were detectable with any methods used. However, in another species *Meriones shawi*, a model of metabolic syndrome and diabetes, that was evaluated again with similar methods^56^, a more severe degeneration was detectable, with a significant decrease in RGC numbers even after 4 months of diabetes. This indicates that the choice of model system used influences – or at least can influence - the rate of retinal damage caused. We have to point out here, that irrespectively of the model examined by us, it seemed to be universally true, that gliosis and outer retinal damage precede detectable ganglion cell loss, which either appeared later^56^, or was completely absent^26,27,78^.

Also, irrespectively of the model system used, there are notable differences reported in animal care. While some groups apply absolutely no treatment throughout the whole observation period^24,26,27,56,78^, the majority of the groups use some kind of insulin substitution^8,25,79,80^ or often even different levels of treatments. Insulin influences weight loss, blood glucose levels, lipid homeostasis among others; and may affect retinal pathology. Insulin receptors are abundant in the photoreceptor layer, and insulin signalling was suggested to have a neuroprotective role – at least for the photoreceptors. Similar role can be assumed (directly or indirectly) for other retinal cell types as well. On the other hand, in a report by Hajna *et al*.^81^ the greatest decrease in retinal thickness was detectable in animals with the highest rate of insulin substitution, so the possible role of insulin therapy in modulating retinal cell loss is still contradictory.

As reviewed above, several OCT studies have reported thinning of the GCL and IPL complex^20–23^ in human diabetic patients even with no or minimal retinopathy – relatively early in the course of the disease. On most OCT scans, however, it is difficult to differentiate (and automatically or manually segment) the GCL and IPL successfully and thus the reported thinning does not necessarily demonstrate GCL loss indeed. In fact, among other factors, it can also be explained by alterations of the IPL, such as thinning or changes in optical properties which can occur due to a disorganized stratification. Correspondingly, thinning of the IPL has been shown to occur in different animal models^8,82^ along with changes of synaptic transmission or glutamate metabolism.

Furthermore, there may be alterations confined to specific sublayers of the IPL in diabetes. The sublayers are formed by the processes of retinal neurons synapsing with their partners in a well-organized and highly specific pattern. Recent publications distinguish 10–11 such strata^83,84^ and evidence suggests that some of these layers are more vulnerable to diabetes than others. A damage or oedema of a specific sublayer may change the overall optical properties and reflectance of the IPL and therefore its thickness on OCT scans.

In the present work, when examining IPL properties in detail, there was no detectable change neither in overall IPL thickness nor in the stratification pattern in ZDF rats, at least with the methods applied.

In summary, hereby we present a detailed assessment of retinal ganglion cell numbers and spatial distribution patterns in ZDF rats, a commonly used T2D model. Analysing and carefully comparing whole mounted retinas with our custom-built algorithm, we excluded any significant RGC loss - even restricted to retinal areas only -, and any major change in staining patterns. The presented algorithm can be used in whole mounts or retinal pieces in all animal models as well as in human samples. In search for an alternative explanation for the GCL and IPL complex alterations on OCT scans, we also assessed and could rule out any significant changes in IPL thickness or stratification pattern in these specimens as well. These results indicate that RGC loss, despite the clinical findings, is probably not one of the earliest of diabetic retinal changes. In ZDF rats examined here and also in other models^26,27,56,78^ RGC loss is preceded by serious outer retinal degeneration, which finding urges for further studies on the potential ganglion cell pathology in diabetes and also underlies that more attention must be paid to the outer retinal alterations in the clinical assessment of early diabetic patients.

## Materials and Methods

### Animal model and tissue preparation

All procedures of the present study were performed in concordance with the Association for Research in Vision and Ophthalmology (ARVO) statement for the Use of Animals in Ophthalmic and Vision Research. The study was approved by the local Ethics Committee for Animal Experimentation of Semmelweis University and by the Animal Health and Animal Welfare Directorate of the National Food Chain Safety Office of the Hungarian State (number of approval: 22.1/1162/3/2010).

Experiments were carried out on ZDF inbred rats, obtained from Charles River Laboratories (Sulzfeld, Germany). Some of the results from the same experiment have already been published^27^. Homozygous recessive males (fa/fa) develop obesity, fasting hyperglycaemia and T2D due to a leptin receptor gene mutation and a special diet (Purina 5008). Homozygous dominant (+/+) and heterozygous (fa/+) genotypes remain normoglycemic and were used as controls in the experiment (ZDF lean). ZDF rats (n = 8) and ZDF lean controls (n = 8) were delivered at the age of 6 weeks, housed in a room with constant temperature (22 ± 2 °C) under a 12–12 h alternating light-dark cycle. Rats were supplied with food and water ad libitum. Body weights and blood glucose levels (Accu-Chek® Sensor, Roche Inc., Mannheim, Germany) were checked regularly throughout the observation period. The animals were euthanized at the age of 32 weeks, after a set of invasive hemodynamic measurements carried out in general anesthesia^85^.

To remove erythrocytes from tissues an *in vivo* perfusion was performed with a total volume of 40 ml oxygenated Ringer solution (37 °C, 8 ml/min) and the animals were decapitated. The eyes were oriented, removed and cut at the *ora serrata*. The cornea, the lens and the vitreous body was removed, and the remaining eyecup was placed into fixative (4% paraformaldehyde diluted in 0.1 M phosphate buffer [PB, pH 7.4]) for two hours at room temperature

### Immunohistochemistry

After fixation, the eyecups were rinsed several times with 0.1 M PB, the retinas were carefully detached with preserved orientation and threated further as whole mounts for RGC counting. The retinas were co-labelled with three different markers:Brn-3a^32,57,86^, NeuN^33^ and RBPMS^34^ that are known to recognize the majority of ganglion cells. The details about the primary antibodies used is given in Table 2. The immunohistochemical procedures were modified from previously published protocols^78^. To block nonspecific staining, retinas were treated with 1% bovine serum albumin diluted in 0.1 M phosphate buffered saline (PBS, pH 7.4), applied overnight at 4 °C, with 0.4% Triton X-100 (Sigma-Aldrich Kft, Budapest, Hungary) added. All primary antibodies used were also diluted in the same solution. Three antibodies were used consecutively, for 48 hours with continuous agitation, and repeated rinsing steps in between (PBS, 10 × 10 minutes). The bound primary antibodies were visualized by species-specific fluorescent dyes (Alexa 488 and Alexa 555 conjugates, 1:200, Thermo Fischer Scientific, Waltham, MA) or in case of RBPMS, biotinylated donkey anti-rabbit (A16039, Thermo Fischer Scientific, Waltham, MA) and streptavidin conjugated Alexa 633 marker. After repeated rinsing steps (PBS) the retinas were flat-mounted and cover slipped with a 1:1 solution of PBS and glycerol.

**Table 2:** Primary antibodies and lectin used in the study.

Antibodies and lectin	Source	Dilution	Host and type	Labelling pattern	Reference
Brn-3a #SC-31984	Santa Cruz Biotechnology Inc., Heidelberg, Germany	1:500	goat polyclonal	retinal ganglion cells	^32, 57^
NeuN Clone:A60 #MAB377	Merck Kft., Budapest, Hungary	1:200	mouse monoclonal	most retinal ganglion cells, some amacrine and displaced amacrine cells	^33^
RNA Binding Protein with Multiple Splicing (RBPMS)	Generous donation of Natik Piri, Jules Stein Eye Institute, University of California, Los Angeles, CA	1:500	rabbit polyclonal	retinal ganglion cells	^34^
choline acetyltransferase (ChAT) #AB144P	Millipore, Billerica, MA	1:200	goat polyclonal	cholinergic amacrine cells, 2 sublayers in the IPL	^41^
calretinin #AB5054	Millipore, Billerica, MA	1:2500	rabbit polyclonal	amacrine cells in the IPL, amacrine and ganglion cells in the GCL, 3 sublayers in the IPL	^39, 40^
lectin from *Arachis hypogaea* (peanut agglutinin lectin, PNA)	Sigma-Aldrich Kft., Budapest, Hungary	5 µg/ml	lectin	interphotoreceptor matrix of all cones, IPL sublayers	^42, 43^

Additionally, retinal sections were also prepared as described in a previous study giving a details about the embedding procedure and sectioning^27^. In this study, only a limited number of sections were used to test the staining characteristics of the antibodies, calculate the number of TUNEL positive elements in the GCL and to estimate possible changes in the thickness and stratification of the IPL. Throughout the study only vertical sections at the level of the optic nerve were used for quantification, from 4 animals per group, and 4 sections per animal. Apoptotic cells in the ganglion cell layer were counted on complete retinal vertical sections using terminal deoxynucleotidyl transferase deoxyuridine triphosphate nick end labelling (TUNEL, *In situ* Cell Death Detection Kit, Fluorescein; Roche Diagnostics, Mannheim, Germany) according to the manufacturer’s recommendations. Immuno- and lectin histochemistry was performed on sections as described previously^26,27^. The bound antibodies were visualized with species specific Alexa conjugates. PNA lectin was used in a biotinylated form and was detected by streptavidin conjugated Alexa-594 dyes. Nuclei were counterstained by DAPI (4,6-diamidino-2-phenylindole, Sigma-Aldrich Kft, Budapest, Hungary).

### Imaging

Images were acquired from retinal sections to test triple ganglion cell labelling, quantifying TUNEL reactions by a Zeiss LSM 780 Confocal System coupled to a Zeiss Axio Imager upright microscope (Carl Zeiss Meditec AG, Oberkochen, Germany) using a 40xobjective, while for determining IPL thickness and stratification a 63x objective was used.

For RGC counting on whole mounted retinas, the complete whole mounted retinas were photographed with the same confocal system, using a 10x objective, the tile image function with 5% overlap and the online stitching function enabled in the Zen 2012 software (Carl Zeiss Meditec AG, Oberkochen, Germany). RGCs were counted manually in 80 ± 14 evenly distributed sample fields across the retina in an area of 0.04 mm^2^

### Isodensity maps

Low resolution images with the position of the counting frames included were created from the whole mounted retinas and were used to detect the outlines of the retina in FIJI^87^ with automated segmentation based on brightness thresholding. The position of the centre of the optic nerve head was selected manually. Local cell density values that were counted manually were ordered to the x and y coordinates of the sampling squares. The outline of the retina, the position of the optic nerve and the data of cell density values with the appropriate positions were imported into MATLAB R2013a (MathWorks Inc., Natick, MA). Since the data points were scattered, in order to approximate the density values for uncounted points, the values were interpolated using the *griddata* function with *cubic* interpolation method, then the results were pasted into the outlines of the appropriate retina and presented in a greyscale map, where the greyscale value (0–255) was equal to the number of RGCs given in 1/sampling unit. These maps were used for all quantitative and qualitative analyses of the assessments detailed in the forthcoming chapters. For all presentation used in this work, the values were expressed as RGCs in 1/mm^2^ and shown in a colour-coded fashion.

During preparation of whole mounts, the retinas were cut at least into four incomplete quadrants to flatten. These incisions should ideally be - but not necessarily are - in the same positions, which makes the point-to-point quantitative comparison of the retinas challenging. There are several methods to correct these artefacts described in the literature^60,62–64^, but most of them require sophisticated mathematical calculations and/or suffer from loss of information due to distortion of the images and data. Therefore, we decided to create a new transformation method, which eliminates gaps, keeps all data point without distortion and does not change their spatial positions relative to each other. Also, the transformation results in an easy to compare, two-dimensional representation of the retina, and requires moderate computing capacity.

The orientation of the retinas was defined by marking the superior position and the location of the optic disc as centre of the whole mount. According to these two parameters, distance from the centre (binned in 1 μm bins) and the angle from the superior direction was ordered to each pixel of the images. Our assumption was on the one hand that the cuts necessary to flatten the retinas do not influence their distance from centre. On the other hand, if we consider pixels on a latitude line (circle with the same distance from the centre), which are separated by the cuts spatially, they still maintain their sequence along the circle. Therefore, the pixels from the same latitude line can be ordered according to their angle to superior position, while empty pixels (pixels of the gaps) can simply be excluded.

The pixels were reordered in a new coordinate system, where the distance from the centre was depicted on the y-axis and rank of angle on the x-axis. It is of note, that each pixel is involved in the resulting graph, none of the pixels were distorted to fit neither on a virtual sphere nor on a polar coordinate system and they are surrounded by pixels with similar distance and angle values which were their natural neighbours before cutting. A schematic drawing of this transformation is given in Fig. 6.

The resulting transformed maps could easily be compared with each other in FIJI. A stack could be created from the transformed maps with the centre (optic nerve head) placed into identical positions. From the stack, a minimum intensity projection creates the overlapping region of the maps (ie: regions that were present in all examined retinas), while a maximum intensity projection estimates the ideal retina without any loss of peripheral regions during preparation. Retinal areas were calculated for the individual retinas, for the overlapping region and for the proposed ideal retina as well allowing us to estimate the “loss during preparation” (the difference between the ideal and individual retinas) of retinas for each individual case and in a group average. Average RGC density values were also calculated and compared for the complete retinas and for the overlapping regions only. For comparison, estimated total RGC numbers were also given for each retina. The data obtained can be analysed statistically.

It is also possible to quantify the regional differences between the transformed retinas. An average intensity projection was created using the overlapping regions of the control retinas only. The density values of the overlapping regions of the individual retinas from both the control and the diabetic groups were subtracted from this average control retina, and the results were shown in a colour-coded fashion and in a form of a histogram which were then compared.

### Measuring IPL thickness and evaluating IPL stratification

IPL thickness was measured on confocal photographs taken 250 and 500 µm away from the optic nerve head in both superior and inferior directions, using a 63x objective (NA: 1.40). The borders of the IPL were selected manually based on the nuclear labelling with DAPI. The thickness was determined at 16 ROIs per photograph on each of the 4 regions using 4 different specimens from each group and 4 sections per specimen.

Confocal photomicrographs co-labelled with ChAT, calretinin and PNA were used to evaluate possible changes in IPL stratifications.

### Statistical analysis

Unless indicated otherwise (in case of some retinal maps) all results were expressed as mean ± SD (standard deviation). Normal distribution was tested by the Shapiro–Wilks method. Two-way analysis of variance (ANOVA) test with repeated measures was applied for body weights and blood sugar level comparisons with Bonferroni’s post hoc test. For RGC density analysis and IPL thickness measurements, given the group sizes of our experiment, the comparison of the control and diabetic groups was performed by two-group exact randomization test^87,88^. In all statistical analyses, P values less than 0.05 were considered significant.

## Supplementary information

Supplementary Information

## Data Availability

The raw datasets generated during the current study and the scripts used during the analysis are available from the corresponding author on reasonable request.
