# Supplementary material for: Detailed Evaluation of Possible Ganglion Cell Loss in the Retina of Zucker Diabetic Fatty (ZDF) Rats
Source: Sci Rep. 2019 Jul 18;9:10463. doi: 10.1038/s41598-019-46879-1 (PMC6639371; doi:10.1038/s41598-019-46879-1)
Supplement: Supplementary file 1 — Supplementary Information [file 41598_2019_46879_MOESM1_ESM.pdf]

Supplementary Figures

Detailed Evaluation of Possible Ganglion Cell Loss in the Retina of Zucker Diabetic Fatty (ZDF) Rats

Rozina I. Hajdú[1,2], Lenke K. Laurik[2], Klaudia Szabó[1], Bulcsú Dékány[1], Zsuzsanna Almási[1], Anna Énzsöly[1,2], Arnold Szabó[1], Tamás Radovits[3], Csaba Mátyás[3], Attila Oláh[3], Ágoston Szél[1], Gábor M. Somfai[2,4#], Csaba Dávid[1#], Ákos Lukáts[1*#]

[1]Department of Anatomy, Histology and Embryology, Semmelweis University, Budapest, Hungary

[2]Department of Ophthalmology, Semmelweis University, Budapest, Hungary

[3]Heart and Vascular Center, Semmelweis University, Budapest, Hungary

[4]Retinology Unit, Pallas Kliniken, Olten, Switzerland

# These authors (G.M.S, Cs.D. and Á.L.) contributed equally to this work.

* Corresponding author:

Email: lukatsakos@gmail.com (Á.L.)

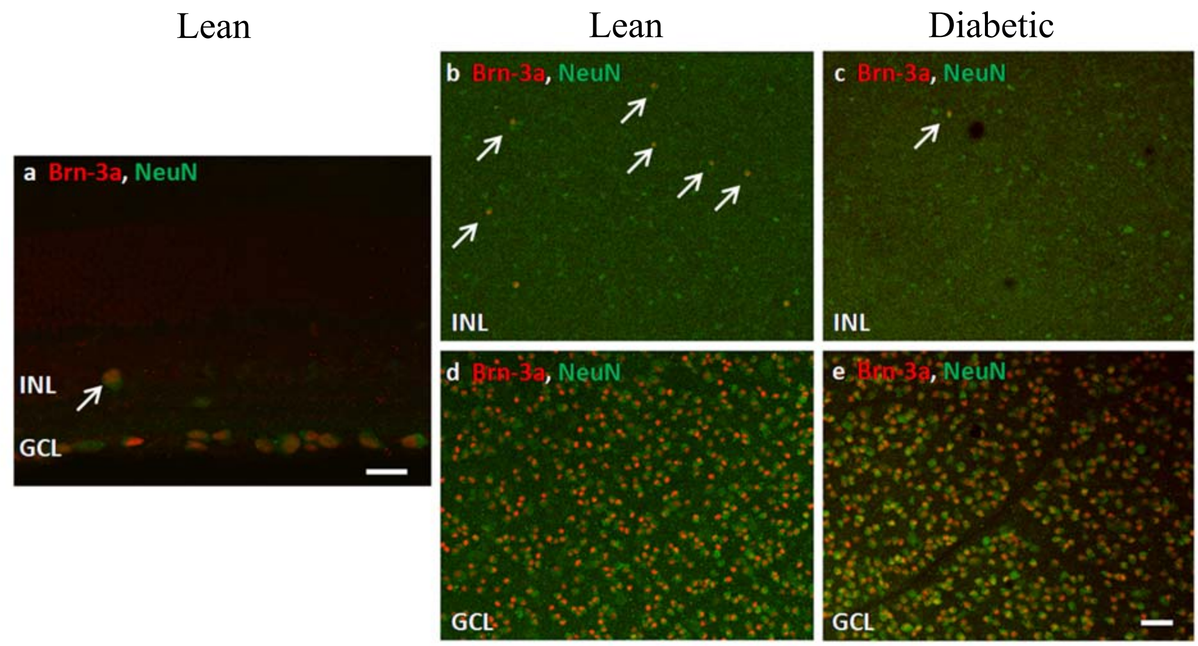

Figure S1. Representative images of retinal ganglion cells in the ganglion cell layer and displaced retinal ganglion cells in the inner nuclear layer. Brn-3a (in red) and NeuN (in green) co-labeled ganglion cells are abundant in the GCL, as demonstrated on vertical sections from lean specimens (a), and on flat mounted retinas of lean (d) and diabetic ZDF rats (e). Relatively large co-labeled displaced ganglion cells may be found only in a much smaller number in the INL, both on sections (a) and on whole mounted retinas from lean (b) and diabetic specimens (c). Some displaced cells are marked by arrows. INL: inner nuclear layer, GCL: ganglion cell layer. *Bar*: 20 µm on figure S1a and 50 µm on Figure S1b-e.

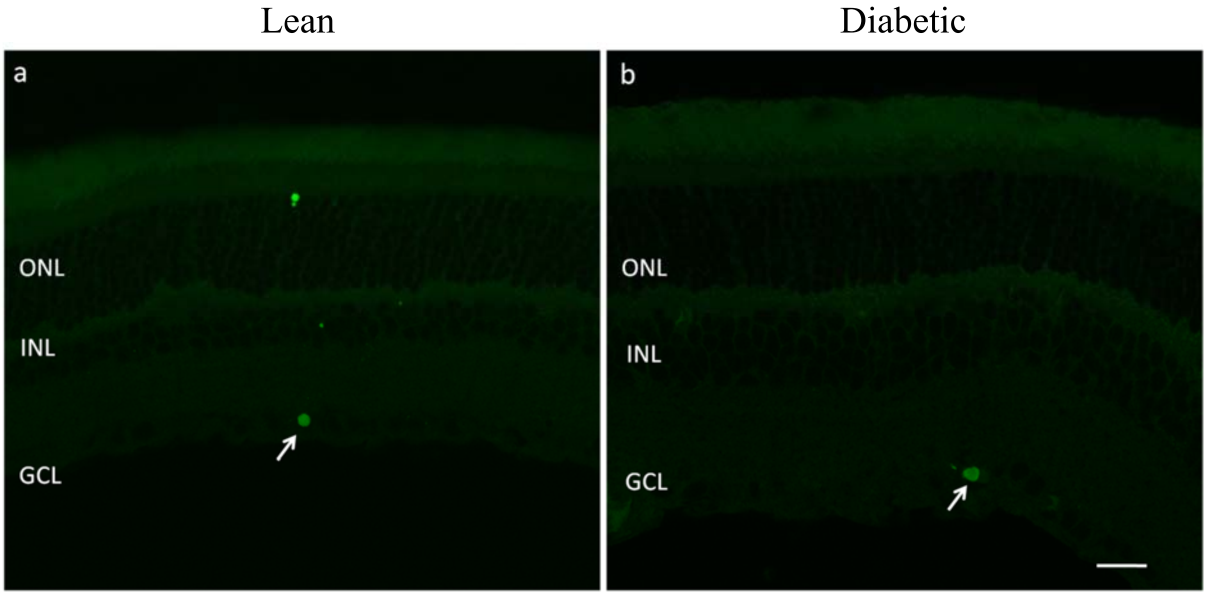

Figure S2. Representative images of TUNEL positive cells in the ganglion cell layer from ZDF lean (a) and diabetic specimens (b). Positive elements are labeled by arrows. ONL: outer nuclear layer, INL: inner nuclear layer, GCL: ganglion cell layer. *Bar*: 20 µm.
